# Automated Quantification of Early Bone Alterations and Pathological Bone Turnover in Experimental Arthritis by *in vivo* PET/CT Imaging

**DOI:** 10.1038/s41598-017-02389-6

**Published:** 2017-05-22

**Authors:** Bianca Hoffmann, Carl-Magnus Svensson, Maria Straßburger, Björn Gebser, Ingo M. Irmler, Thomas Kamradt, Hans Peter Saluz, Marc Thilo Figge

**Affiliations:** 1 Departemet Cell and Molecular Biology, Leibniz-Institute for Natural Product Research and Infection Biology, Hans-Knöll-Institute, Beutenbergstr. 11a, 07745 Jena, Germany; 2 Friedrich Schiller University, Fürstengraben 1, 07743 Jena, Germany; 3 Applied Systems Biology, Leibniz-Institute for Natural Product Research and Infection Biology, Hans-Knöll-Institute, Beutenbergstr. 11a, 07745 Jena, Germany; 4 Transfer Group Anti-infectives, Leibniz-Institute for Natural Product Research and Infection Biology, Hans-Knöll-Institute, Beutenbergstr. 11a, 07745 Jena, Germany; 5 Institute of Immunology, Jena University Hospital, Leutragraben 3, 07743 Jena, Germany

## Abstract

The assessment of bone damage is required to evaluate disease severity and treatment efficacy both in arthritis patients and in experimental arthritis models. Today there is still a lack of *in vivo* methods that enable the quantification of arthritic processes at an early stage of the disease. We performed longitudinal *in vivo* imaging with [^18^F]-fluoride PET/CT before and after experimental arthritis onset for diseased and control DBA/1 mice and assessed arthritis progression by clinical scoring, tracer uptake studies and bone volume as well as surface roughness measurements. Arthritic animals showed significantly increased tracer uptake in the paws compared to non-diseased controls. Automated CT image analysis revealed increased bone surface roughness already in the earliest stage of the disease. Moreover, we observed clear differences between endosteal and periosteal sites of cortical bone regarding surface roughness. This study shows that *in vivo* PET/CT imaging is a favorable method to study arthritic processes, enabling the quantification of different aspects of the disease like pathological bone turnover and bone alteration. Especially the evaluation of bone surface roughness is sensitive to early pathological changes and can be applied to study the dynamics of bone erosion at different sites of the bones in an automated fashion.

## Introduction

Rheumatoid Arthritis (RA) is one of the most common autoimmune diseases with a prevalence of up to 1% in developed countries^1^. Main characteristics of RA include synovitis and painful joint swelling in the early stages followed by subsequent bone erosion, which leads to loss of joint function and life quality. Early diagnosis is crucial for the success of disease-suppressing anti-inflammatory treatments and there is a window of opportunity for early therapeutic intervention^2–4^. Consequently, sensitive diagnostic methods are inevitable to increase treatment success and to monitor treatment efficacy. Besides early diagnosis and continued monitoring, it is also important to understand the underlying mechanisms that lead to progressive, erosive RA. Animal models have not only provided insights into molecular mechanisms but also allow the development and preclinical studying of new diagnostic methods and therapeutic approaches^5–7^.

For a long time plain radiography (x-ray) in humans and histopathological examination in animals have been the gold-standards to assess arthritic processes. Their major drawbacks are insufficient resolution, poor visualization of complex structures and in case of histopathological analysis the high number of laboratory animals to be sacrificed for tissue analysis. In contrast, ultrasound, magnetic resonance imaging (MRI), computed tomography (CT) and positron emission tomography (PET) are minimal-invasive and allow, similar to x-ray, for longitudinal *in vivo* studies, whereas CT and MRI yield much higher resolution and PET offers insights into metabolic processes^8^.

We have shown earlier that the PET tracers [^18^F]-fluorodeoxyglucose ([^18^F]-FDG) and [^18^F]-fluoride are feasible to assess joint inflammation and pathological bone metabolism in a model of glucose-6-phosphate isomerase (G6PI)–induced arthritis^9–11^. CT has also widely been used to quantify pathological changes of the bones in experimental models as well as RA patients^12–16^. Bone erosion occurs already early in the course of RA^3, 12^ and, therefore, many studies aim to quantify this erosive process. Silva *et al*.^17^ proposed an approach based on the quantification of bone surface roughness. As the erosion and, thereby, the increasing roughness of the surface should be a precursor of visually detectable lesions, this method seems to be very promising for early and sensitive quantification of bone destruction. Nevertheless, this approach has never been used for further studies of experimental arthritis models, which could be due to a crucial scale parameter that has to be defined for each study and can potentially influence the results to a great extent.

In this work, we used *in vivo* [^18^F]-fluoride PET/CT imaging to quantify pathological bone metabolism and bone destruction in a model of G6PI-induced arthritis in mice. For the first time, we evaluated the bone surface roughness longitudinally in this experimental model and compared these results to non-immunized control animals. In contrast to Irmler *et al*.^10^, we used a roughness quantification based on surface triangulation, which is minimally affected by bone growth and yielded a high sensitivity to bone destruction in the early stage of the disease. These two aspects were lacking in the surface area representation of roughness used by Irmler *et al*.^10^. Additionally, we investigated the progression of bone surface roughness at the endosteal and periosteal sites of cortical bone separately as well as different spatial scales on which the roughness occurs. This concept allowed us to detect unequal impairment of endosteal and periosteal surfaces by experimental arthritis without injuring the animals. The calculation of bone surface roughness was implemented as a fully automated image analysis pipeline, which also includes automated segmentation of the regions of interest and therefore allows for high-throughput studies that are free of user bias. Finally, quantitative results from PET and CT as well as clinical scoring were compared to each other by correlation analysis in the present study.

## Material and Methods

### Glucose-6-phosphate isomerase–induced arthritis

Female inbred DBA/1 mice (weight 16.2–19.5 g; University Hospital, Jena, Germany) were housed under standard conditions (temperature: 20 ± 2 °C, humidity: 50 ± 10%, light/dark cycle: 12/12 h) in groups of 3 to 4 animals in individually ventilated cages, and fed with normal mouse chow and water *ad libitum*. All animals were cared for in accordance with the principles outlined by the European Convention for the protection of vertebrate animals used for experimental and other scientific purposes. Experiments were in compliance with the German animal protection law and were approved by the Federal State Authority of Thuringia and ethics committee (permit Reg.-Nr. 02–001/14). Experimental arthritis was induced as described earlier^11^ at an age of 10 to 13 weeks in 10 animals that were picked at random. In brief, mice were immunized subcutaneously with 400 μg of recombinant human G6PI emulsified in complete Freund’s adjuvant (Sigma-Aldrich, Taufkirchen, Germany). Clinical scoring of arthritis manifestation was performed macroscopically. Swelling and erythema of wrist and ankle joints, metacarpophalangeal (MCP) and metatarsophalangeal (MTP) joints in each paw was graded from 0 to 3 as established by Irmler *et al.* ^10^. Representative images of mice paws for the respective scores are shown in Supplementary Fig. S1. The cumulative clinical score for each paw was calculated as the sum of the scores for wrist/ankle joint, MCP/MTP joint and digits/toes.

### Positron emission tomography/Computed tomography *in vivo* imaging

*In vivo* imaging was performed with a multimodal Siemens Inveon Small Animal PET/CT system (Siemens Healthcare Medical Imaging). The PET modality has radial, axial and transaxial resolutions of 1.5 mm at the center of the field of view^18^. We performed PET acquisitions with a coincidence timing window of 3.4 ns and an energy window of 350–650 keV. PET acquisitions, each with duration of 20 minutes, were started 35 minutes after injection of [^18^F]-fluoride in 0.9% sodium chloride solution with an activity of approximately 10 MBq into the tail vein. 3D PET images were reconstructed with three-dimensional ordered subset expectation maximization/maximum *a priori* algorithm and CT-based attenuation correction. The CT modality consists of a cone beam x-ray micro–CT (µCT) source with a focal spot size of 50 μm and a built-in 0.8 mm carbon fiber filter and a 3,072 × 2,048–pixel x-ray detector. The μCT acquisition protocol for high resolution scans of hind paws used 2,048 × 2,048–pixel axial-transaxial resolution, magnification parameter *med-high*, 80 kV at 500 μA, 3500 ms exposure time, total rotation of 360° and 360 projections per scan. CT images were reconstructed using a Shepp–Logan filter and cone-beam filtered backprojection with a pixel resolution of 14.609 µm. The animals were anesthetized with 3% isoflurane (Deltaselect, Dreieich, Germany) vaporized in oxygen (1.5 l/min) in an external anesthesia chamber prior to the PET/CT scans and kept under anesthesia throughout the scans with 1.5% isoflurane vaporized in oxygen (1.5 l/min) to prevent animal movement. Anesthesia was monitored by measuring the respiratory frequency and the body temperature was kept at 37 °C by using a heating pad. Imaging was performed longitudinally before immunization (day −4) and at different time points (days 10, 14, 18/20, 24 and 35) of acute and chronic arthritis with immunized mice (n = 10) and non-immunized (n = 6) controls. The overall scanning time for one animal at one time point was approximately 55 min.

### Quantification of pathophysiological bone metabolism

The quantitative uptake of [^18^F]-fluoride as a measure of pathophysiological bone metabolism in fore and hind paws of the mice was calculated with Siemens Inveon Research Workplace 4.0 software based on fused PET/CT images. Guided by the CT images spherical (ellipsoids) volumes of interest (VOIs) were placed manually around the bone and joint structures of fore and hind paws, respectively. A representative cross-sectional slice with overlaid VOI boundaries is shown in Supplementary Fig. S2. Each VOI was then thresholded by a value of 40% to the pixels with the highest standard uptake value (SUV; g/ml), which describes the incorporation of [^18^F]-fluoride into the bone and is defined as:

1 $${\rm{SUV}}=\frac{{\rm{average}}\,{\rm{activity}}\,{\rm{in}}\,{\rm{VOI}}}{{\rm{injected}}\,{\rm{activity}}}\,{\rm{body}}\,{\rm{weight}}$$

Reasonable threshold values were found to range from approximately 30% to 50%, while the threshold was not very sensitive to changes within this range (see Supplementary Fig. S2). Based on this segmentation the mean SUV and standard deviation in each VOI was calculated automatically by the software.

### Automated preparation of VOIs based on μCT images

**Figure 1:**
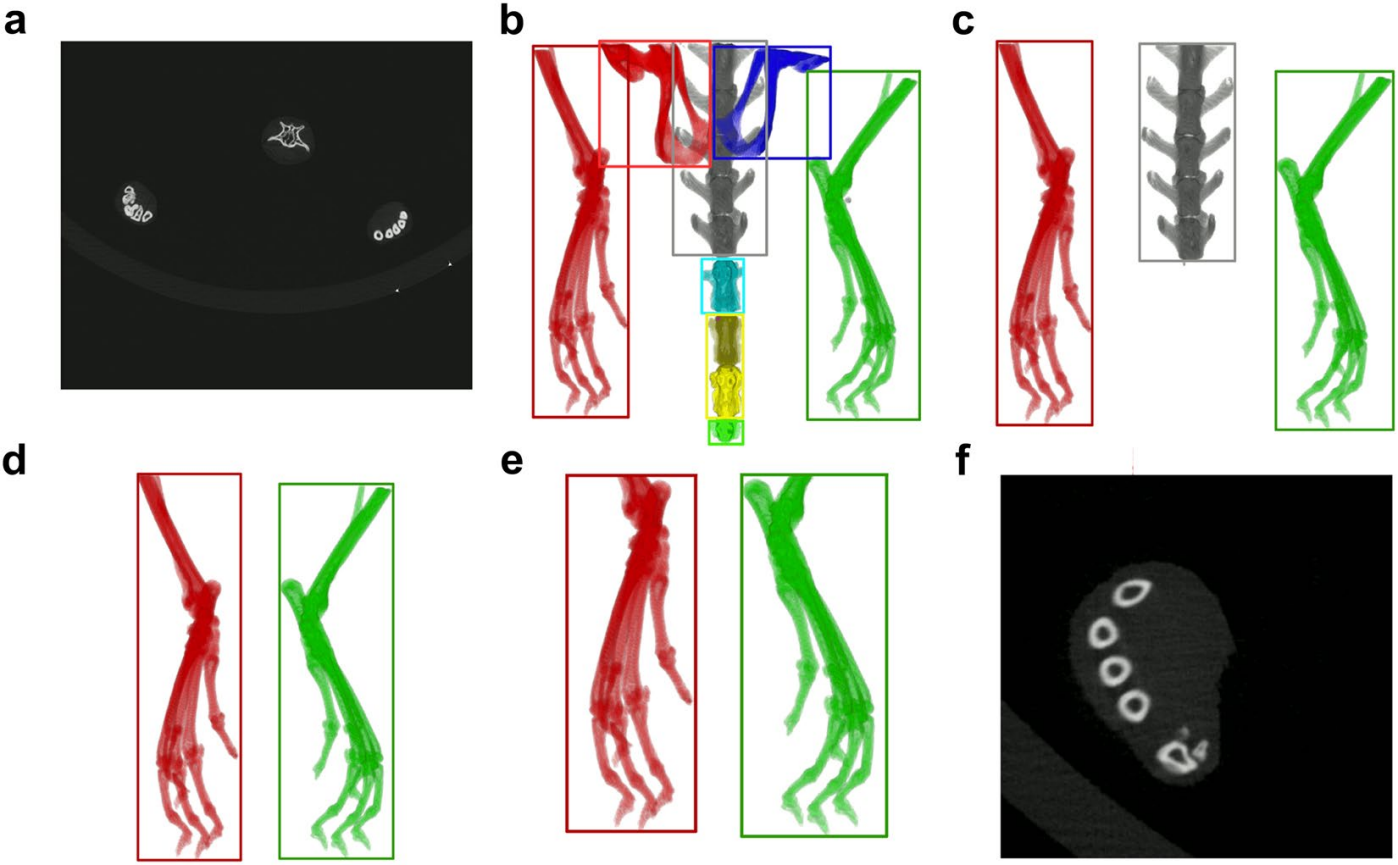
Preparation of defined μCT VOIs containing left and right hind paws. 16-bit image stacks (**a**) were converted to 8-bit grayscale, down sampled by factor 4 and binarized via intensity thresholding to foreground and background pixels. Bounding boxes represent individual, connected structures of foreground pixels which are shown here as volume rendering (**b**). All bounding boxes containing less foreground pixels than a certain threshold were discarded (**c**), as well as bounding boxes that are not located at the most left and most right regions of the image stack (**d**). The two bounding boxes representing left and right hind paw were shrunk to a defined region, which was determined by the end of the calcaneus bone (**e**) and the obtained VOIs were cut out of the original stack (**f**, shown here: left hind paw).

For further analysis high resolution μCT images were automatically cut to VOIs including only left and right hind paw, respectively (Fig. 1). To this end, each μCT image stack (Fig. 1a) was converted from 16-bit to 8-bit grayscale with intensity values between 0 and 255 and down-sampled by factor 4 in order to speed up the algorithm. The image stack was binarized by thresholding with an intensity value of 75 into foreground (bony/cartilage structures) and background (surrounding tissue and air) pixels. The fairly low threshold value was optimized in regard to include only high density pixels representing bone material and to ensure that anatomically connected regions are not split. Based on this binarization 8-connected foreground pixels in the 3D space of the image stack were found and these objects were from now on represented as the coordinates and size of surrounding bounding boxes (Fig. 1b). Smaller objects like pelvic bones were excluded from further processing by considering exclusively bounding boxes containing more than 85,000 foreground pixels as potential candidates to represent left or right hind paw (Fig. 1c). Furthermore all bounding boxes that did not appear in a predefined section in z-space (*z*_*min*_ < 300 or *z*_*max*_ *-z*_*min*_ < 300) were excluded (Fig. 1d). The bounding boxes that represent left and right hind paw were found based on their coordinates in x-, y- and z-space, as the two paws usually appear in the lower left and right regions of the image stack for our image acquisition protocol. The endpoint of the hind paws was defined where the tibia enters the tarsocrural joint and the calcaneus starts to appear in the image stack. This position was found by the typical pattern of the number of connected objects in each slice in z-direction (Supplementary Fig. S3). According to this new endpoint the bounding boxes were shrunk in z-direction (Fig. 1e), then upsampled by factor 4 and some additional space was added in each direction (x: 2 × 20 pixels, y: 2 × 20 pixels, z: 2 × 10 pixels). The resulting region was cut out of the original high-resolution CT image (Fig. 1f). The automated VOI preparation algorithm was implemented in Java using the ImageJ library v1.49b^19^.

### μCT-based assessment of VOI roughness

Based on the prepared μCT VOIs triangulated surface meshes were reconstructed with the marching cubes (MC) algorithm^17, 20^. The intensity threshold for the MC algorithm was obtained individually for each VOI by Otsu’s method^21^ to compensate for variations in the image intensity, caused by the imaging modality itself. In order not to lose resolution, the grid size for MC was chosen to be equal to one pixel. Additionally, we corrected the surface meshes for ambiguities according to Cignoni *et al*.^22^. Potentially duplicated vertices and remaining artifacts, which were not representing parts of the hind paws, were removed automatically from surface meshes by a script using MeshLab software 64-bit v1.3.3^23^. For the removal of artifacts a cutoff value of 1,900,000 triangles was chosen as the surface meshes of the hind paws comprised 2,320,000 triangles on average, ranging from 1,930,000 to 3,194,000 triangles. For following comparison, three different surface reconstructions of each VOI were created:

(i) complete surface (*cs*): comprises the whole surface, as described above
(ii) outer surface (*os*): comprises only the periosteal bone surface
(iii) inner surface (*is*): comprises only the endosteal bone surface

**Figure 2:**
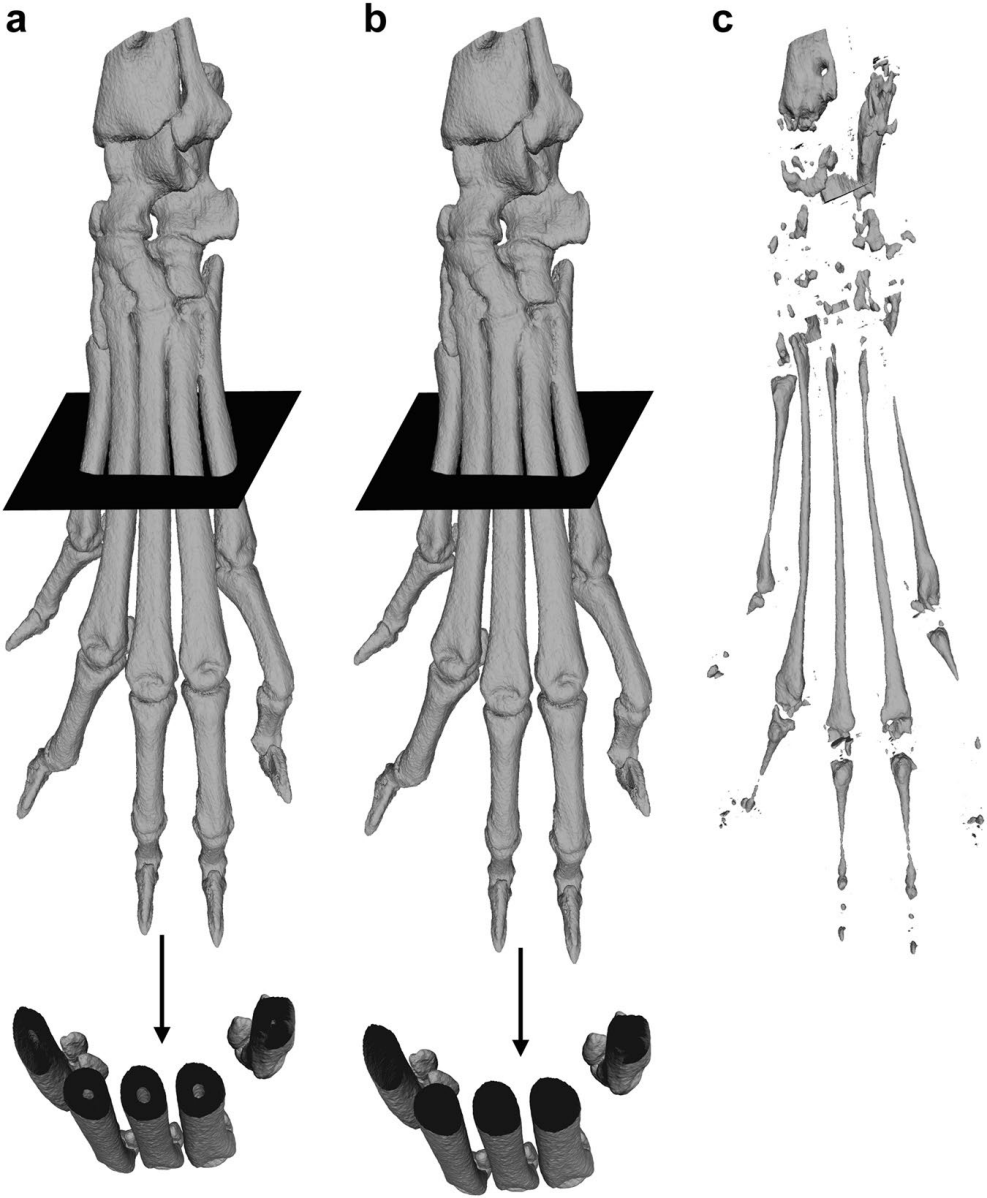
Visualization of complete, outer and inner cortical bone surface meshes. (**a**) The complete cortical bone surface mesh comprises the periosteal surface of the cortical bone and the endosteal surface which is separating the cortical substance from the medullary cavity and can be seen in the cross-sectional view at the bottom. (**b**) The surface mesh of the outer cortical bone surface only consists of the periosteal surface of the cortical substance. The endosteal surface is not part of this mesh, as can be seen in the corresponding cross-sectional view. (**c**) The surface mesh of the inner cortical surface only comprises the endosteal surface that separates the cortical substance from the medullary cavity.

These three surface reconstructions are individually visualized in Fig. 2. The roughness of the three surface reconstructions of each paw was in principal calculated as described by Silva *et al*.^17^. In brief, for each triangle in a surface mesh a circular vicinity of adjacent triangles was defined by a roughness radius *r* (Supplementary Fig. S4). The angles between each adjacent triangles’ normal vector and the current triangle’s normal vector was computed and the mean angle was obtained. The angle information of all surface meshes of the control data (before immunization) was compiled into a composite histogram. A threshold angle, which discriminates between smooth and rough regions was obtained by fitting a probability density function (PDF) to this histogram. While Silva *et al*. used a gamma PDF, we decided to use a lognormal PDF (equation (2)), as it is computationally more efficient and fits better to the composite histogram of our VOIs (Supplementary Figs S5, S6 and S7).

2 $$p(x)=\frac{1}{x\sigma \sqrt{2\pi }}{e}^{-\frac{{(\mathrm{ln}x-\mu )}^{2}}{2{\sigma }^{2}}}$$

The threshold angle, *T*, was calculated as described by Silva *et al*. as the mean plus 2 standard deviations of the PDF:

3 $${\mu }_{p(x)}={e}^{\mu +\frac{{\sigma }^{2}}{2}}\,{\rm{and}}\,{\sigma }_{p(x)}=\sqrt{({e}^{{\sigma }^{2}}-1){e}^{2\mu +{\sigma }^{2}}}$$

4 $$T={\mu }_{p(x)}+2{\sigma }_{p(x)}$$

Roughness for the complete surface, *R*_*VOI,cs*_, the outer surface, *R*_*VOI,os*_, and the inner surface, *R*_*VOI,is*_, was calculated for each VOI by adding up the frequencies of all angles of the respective surface mesh that were greater than *T*. To assess the scale of roughness on which bone erosion occurs, we calculated *R*_*VOI,cs*_, *R*_*VOI,os*_ and *R*_*VOI,is*_ for 6 different roughness radii *r*: 1, 3, 5, 10, 15 and 20, which correspond to ~15, 44, 73, 140, 219 and 292 μm, respectively. The values of *r* were chosen so that they scan from the lowest reasonable radius in agreement with the approximate pixel size resolution to one that covers a big area without wrapping around any bones.

### μCT-based assessment of VOI volume

Based on the μCT image stacks and corresponding thresholds that were used for the MC algorithm, we computed the VOI volume *V*_*VOI*_ using Fiji software v1.49b and the included 3D Objects Counter plugin^24^. *V*_*VOI*_ is the number of connected foreground pixels of the biggest identified 3D object multiplied by the image voxel size of 3.11∙10^−6^ mm^3^.

### Statistical Analysis

Differences between groups were evaluated with the non-parametric Mann-Whitney *U* test (two-tailed) and significant differences were accepted for *p* < 0.05. The results are reported as mean ± standard deviation. For comparison of different roughness radii *r* the ratio of roughness (*μ*_*AR/CO*_, equation (5)) was calculated by dividing the mean roughness of the arthritic group, *μ*_*AR*_, by the mean roughness of the control group, *μ*_*CO*_. The standard deviation *σ*_*AR/CO*_ for *μ*_*AR/CO*_ was calculated according to propagation of uncertainty:

5 $${\mu }_{AR/CO}=\frac{{\mu }_{AR}}{{\mu }_{CO}}$$

6 $${\sigma }_{AR/CO}=|{\mu }_{AR/CO}|\sqrt{{(\frac{{\sigma }_{AR}}{{\mu }_{AR}})}^{2}+{(\frac{{\sigma }_{CO}}{{\mu }_{CO}})}^{2}}$$

Statistical differences between *μ*_*AR/CO*_ for different groups were evaluated by bootstrapping and significance was assumed when the 95% confidence intervals of *μ*_*AR/CO*_ of two groups did not overlap. In the correlation analysis the Spearman correlation coefficient was calculated and significance was accepted for *p* < 0.05. Statistical analysis was performed in R^25^ using the *stats* package for correlation analysis.

An overview of the study design and image analysis pipeline, including the relevant processing steps and software used are given in the Supplementary Fig. S8. The Java implementation of the image analysis pipeline and raw data is available from the authors upon request. The number of data points used for statistical analysis for each experiment can be found in the Supplementary Table S1.

## Results

### Evaluation of clinical score in progressing murine arthritis

**Figure 3:**
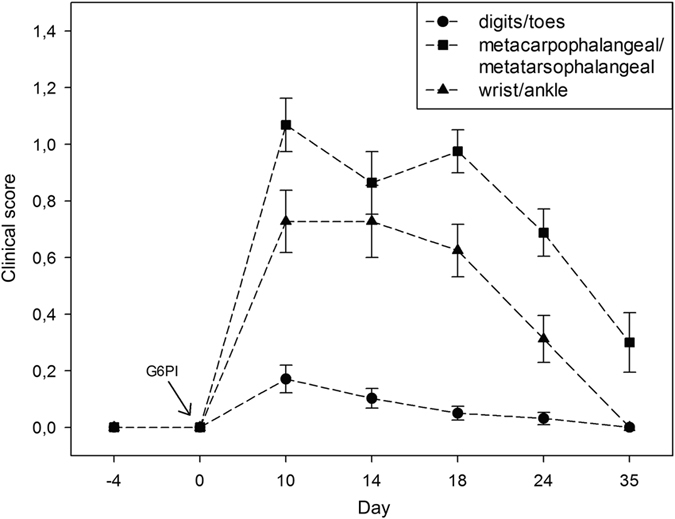
Course of macroscopically visible arthritis before and after immunization with G6PI. The maximum of redness and swelling was observed at day 10 and the clinical score remained at a high level until day 18. In the remitting phase the clinical score subsequently declined. The visible inflammation mainly manifested in MCP and MTP joint regions. Error bars denote the standard error of the mean clinical score.

The immunization of DBA/1 mice with G6PI led to severe symmetric polyarthritis of the small joints of fore and hind paws. First macroscopic signs of the disease could be observed at day 9 after immunization and most animals showed severe inflammation at day 10, indicated by marked swelling and redness. Throughout the acute stage from day 14 to day 18 the clinical score remained high, followed by subsequent decrease until day 35 (Fig. 3). Especially MCP and MTP joint regions showed severe signs of G6PI-induced arthritis, while wrist and ankle joints yielded overall lower clinical scores. At day 35 clinical signs of inflammation were not visible anymore in wrist/ankle joints and digits/toes.

### Quantification and localization of pathophysiological bone metabolism by [^18^F]-fluoride PET

**Figure 4:**
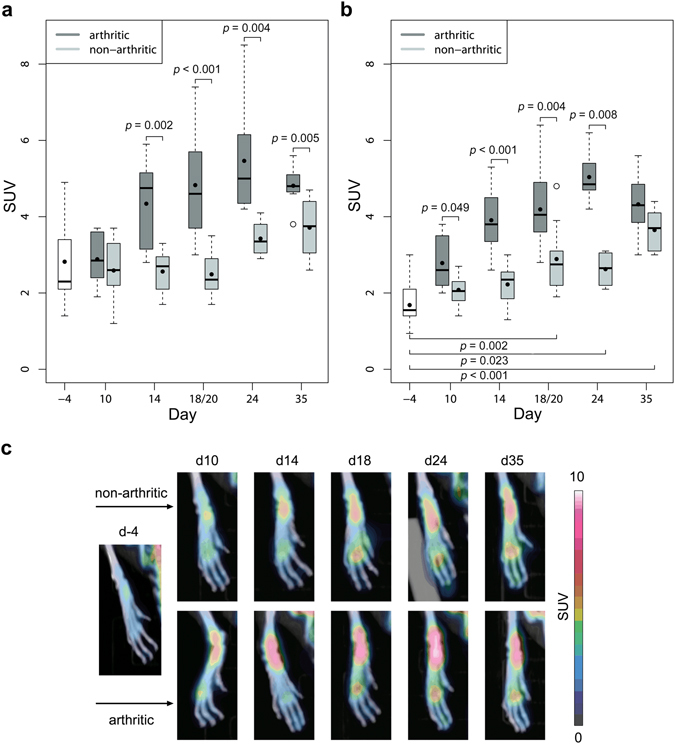
Quantification and localization of pathophysiological bone metabolism in G6PI-induced arthritis. (**a**) Compared to healthy non-arthritic animals the SUV revealed significant increase of [^18^F]-fluoride uptake in fore paws of arthritic mice starting at day 14 after immunization while tracer uptake of healthy animals did not change compared to day −4. (**b**) Tracer uptake in arthritic hind paws was significantly increased already at day 10 after immunization and healthy animals also showed increased uptake in the hind paws starting at day 20 compared to day −4. (**c**) While [^18^F]-fluoride uptake in the hind paws was evenly distributed at day −4, the tracer accumulated predominantly in tarsometatarsal and MTP joint regions at later time points in both, healthy and arthritic animals.

Pathophysiological bone metabolism was quantified by the calculation of the SUV in fore and hind paws, describing the amount of incorporated tracer in these VOIs. The measured amount of overall injected [^18^F]-fluoride was 10.56 ± 1.85 MBq and PET image acquisition was started after 34.92 ± 0.57 min. Some animals had to be excluded from further analysis at some time points due to partially paravenous injected tracer, resulting in 6 to 14 fore and hind paws that could be examined in each group (control, arthritic) for each time point. Before immunization mean SUVs in fore and hind paws were 2.82 ± 0.58 and 1.68 ± 1.14, respectively. Fore paws of arthritic animals showed significantly increased tracer uptake starting at day 14 after immunization (*p* = 0.002, SUV 4.34 ± 0.79) with a maximum increase of 93% compared to day −4 at day 24 and declining tracer incorporation at day 35 (Fig. 4a). Hind paws of arthritic animals showed significantly increased tracer uptake already at day 10 after immunization (*p* = 0.049, SUV 2.78 ± 0.83). The highest average uptake in arthritic hind paws could also be observed at day 24 after immunization with an increase of 207% compared to day −4 (Fig. 4b). While tracer incorporation in arthritic fore and hind paws follows the same trend, significantly increased tracer uptake of healthy control animals could only be observed in hind paws starting at day 20 (*p* = 0.002, SUV 2.89 ± 0.62) with a maximum increase of 122% at day 35. In general, fore paws in both groups showed higher [^18^F]-fluoride uptake than hind paws, while the onset of increased tracer uptake appeared earlier in hind paws and reached higher percental increase. Although [^18^F]-fluoride is a bone seeking tracer that is incorporated also into the bones of healthy animals, arthritic animals showed considerably higher tracer uptake in fore and hind paws after arthritis onset.

The exact localization of [^18^F]-fluoride uptake in hind paws revealed that the tracer is uniformly distributed before immunization, but accumulates especially in tarsometatarsal and MTP joint regions at later time points in control and arthritic animals (Fig. 4c).

### Accuracy of µCT VOI preparation

The automated preparation of VOIs based on µCT images was performed for 95 datasets, each containing the two hind paws, and yielded an accuracy of 92.1%. VOIs were identified and cut correctly for 175 of the 190 paws. In 5 cases the paw could not be identified and in another 10 cases the VOI was cut falsely. For these datasets VOIs were created manually if the image was free of motion artifacts.

### Quantification of VOI roughness in progressing murine arthritis

**Figure 5:**
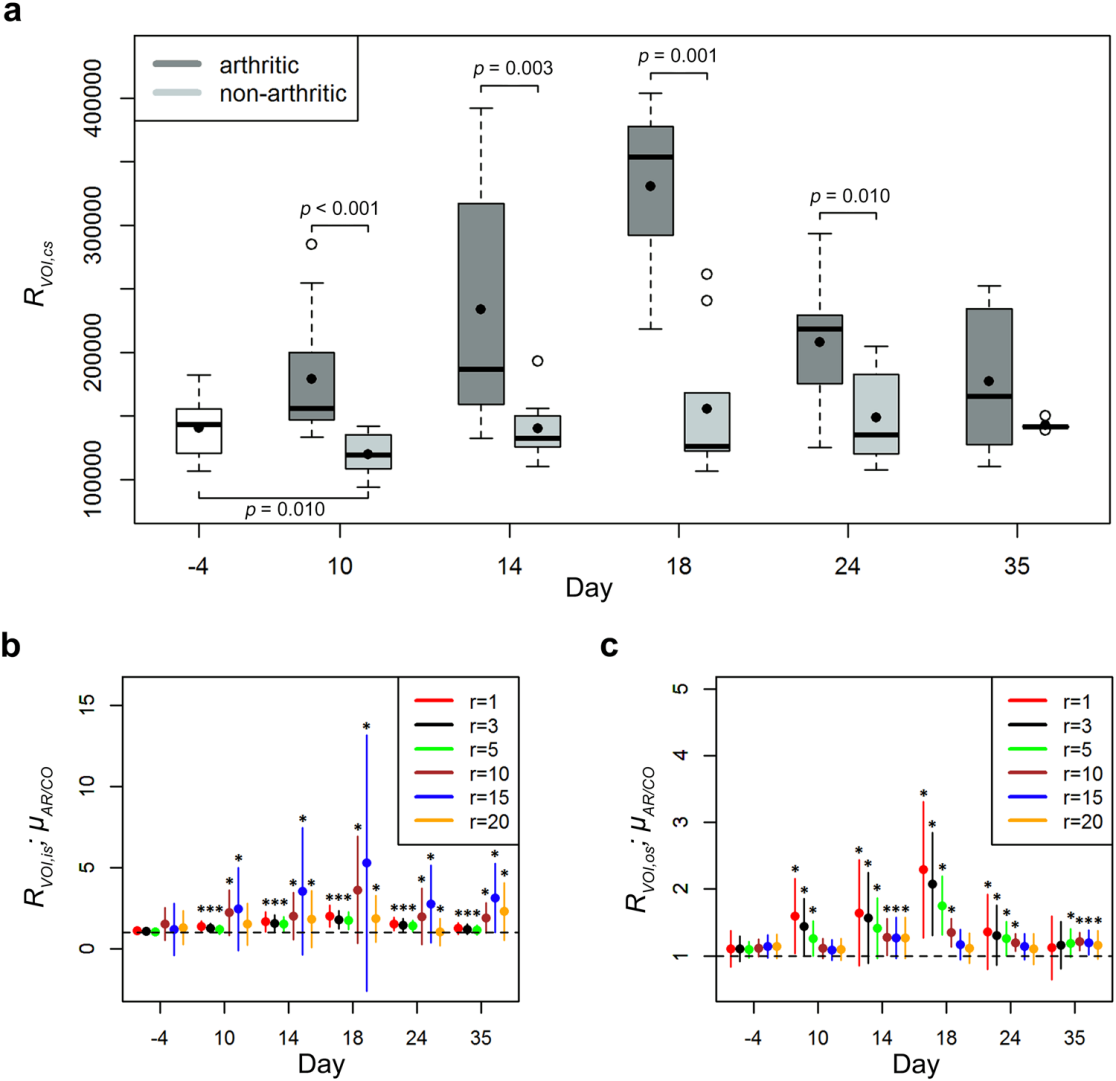
VOI roughness of arthritic and healthy non-arthritic animals for different time points and roughness radii. (**a**) The hind paws of arthritic animals showed significantly increased VOI roughness of the complete surface at days 10, 14, 18 and 24 after immunization compared to healthy animals (roughness radius *r* = 3). For the group of healthy mice roughness was significantly decreased at day 10 compared to the control measurements of all animals before immunization. (**b**) The VOI roughness of the inner surface was more pronounced for roughness radii *r* = 10 and *r* = 15, while the VOI roughness of the outer surface occurred predominantly on a small spatial scale for roughness radii *r* = 1 to *r* = 5 (**c**). The increase of VOI roughness of arthritic animals reached its peak at day 18 after immunization and subsequently declined until day 35 in all cases.

**Figure 6:**
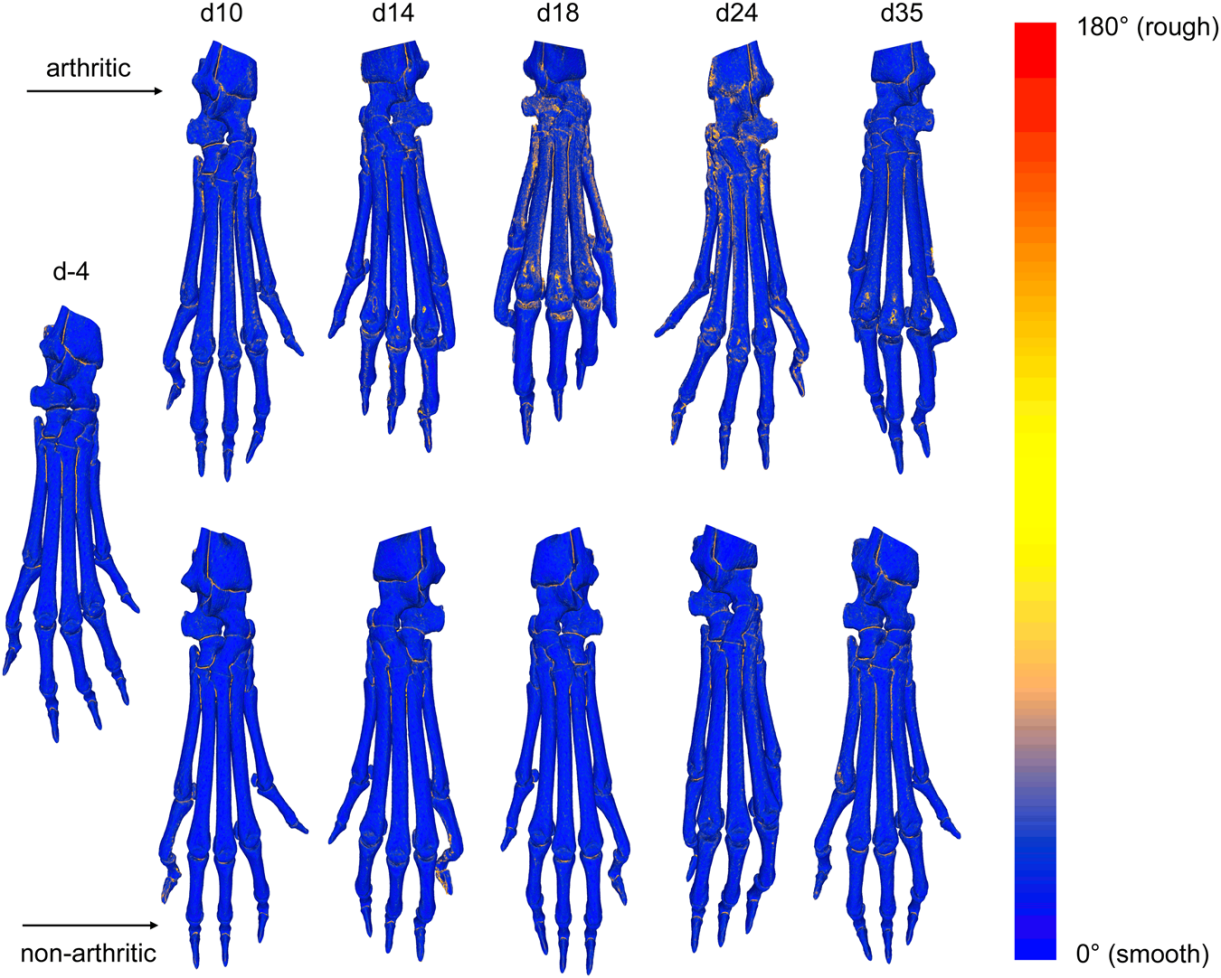
Visualization of bone surface roughness in arthritic (top row) and non-arthritic (bottom row) hind paws over time. The roughness of the bone surface is visualized by a color gradient ranging from blue (smooth surface) to red (rough surface) and shown here for representative hind paws for the different time points. First signs of erosion get visible at day 10 after immunization near to the MTP joints in arthritic animals. The roughness is further increasing until day 18 where large parts of the bone surface are affected which is indicated by the orange color. At later time points the roughness is declining again and increased surface roughness is restricted to the regions near to the MTP joints at day 35. In contrast, the bone surface of non-arthritic animals is constantly smooth over time.

Bone destruction caused by G6PI-induced arthritis was evaluated by calculation of the VOI roughness, which captures erosive processes at the surface of bony and other high density structures of the hind paws. VOI roughness of the complete surface (*R*_*VOI,cs*_) of arthritic animals was significantly increased compared to healthy animals at days 10 to 24 after immunization (Fig. 5a). The maximum increase was reached at day 18 after immunization with an increase of 113% compared to the control animals and 135% compared to day −4. *R*_*VOI,cs*_ of control animals was significantly decreased at day 10 after immunization in comparison to the measurements of all animals at day −4 (*p* = 0.01). In the past, histological studies and manual assessment of radiographs revealed that the endosteal and periosteal surfaces of cortical bone are impaired unequally by experimental arthritis and that bone formation and bone erosion are not uniformly distributed throughout these surfaces^26–29^. In order to investigate these differences by non-invasive µCT imaging we calculated the bone surface roughness separately for the inner (endosteal) bone surface and the outer (periosteal) bone surface for different roughness radii *r*, under the assumption that bone erosion and bone formation cause roughness on different spatial scales. For roughness of the inner surface, *R*_*VOI,is*_, the ratio *μ*_*AR/CO*_ was significantly increased for almost all examined roughness radii *r* at all time points after immunization compared to day −4, but mostly pronounced between *r* = 10 and *r* = 20 (Fig. 5b). The maximum increase of 351% was reached at day 18 for *r* = 15. In contrast, *μ*_*AR/CO*_ of the outer surface, *R*_*VOI,os*_, was predominantly increased for smaller roughness radii in the range of *r* = 1 to *r* = 5 at days 10 to 24 and larger roughness radii in the range of *r* = 5 to *r* = 20 at day 35 after immunization (Fig. 5c). The maximum increase of 107% was also found at day 18 after immunization, but for *r* = 1. The time course of roughness progression in arthritic and non-arthritic animals is shown in Fig. 6 by color-coded surface meshes of representative hind paws.

### Quantification of VOI volume in progressing murine arthritis

**Figure 7:**
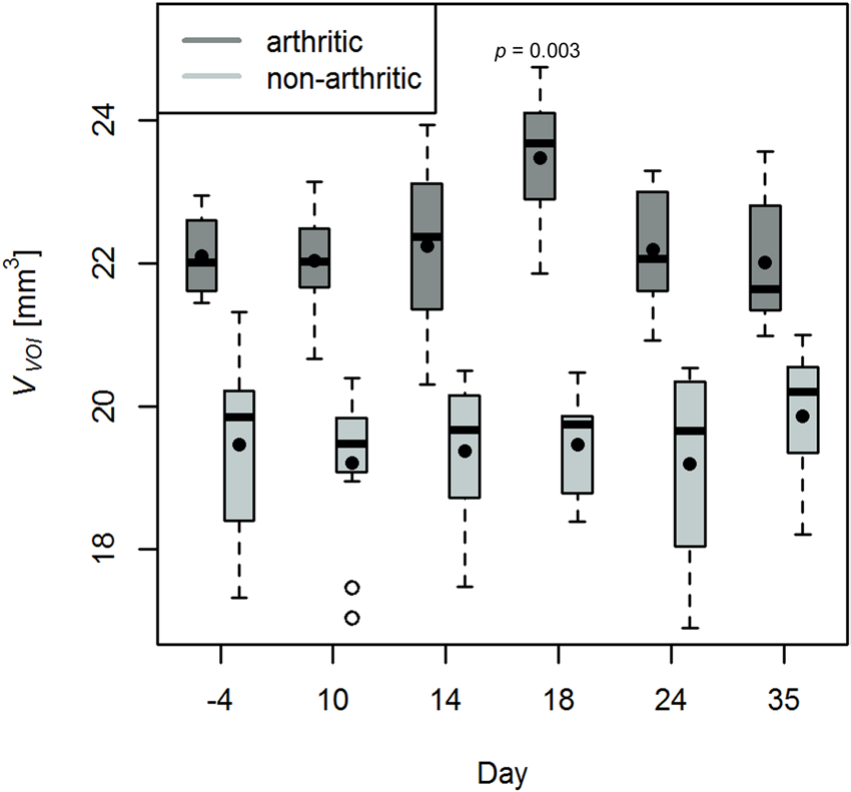
VOI volume for arthritic and healthy non-arthritic animals. The VOI volume of the group of arthritic animals was overall larger compared to the group of healthy animals, but a significant increase of VOI volume over time could only be observed for arthritic animals at day 18 after immunization.

The change of VOI volume in the course of G6PI-induced arthritis for arthritic and healthy animals is shown in Fig. 7. Compared to day −4 the arthritic animals showed a statistically significant increase in *V*_*VOI*_ at day 18 after immunization with a total increase of 6% (*p* = 0.003). Such a difference could not be observed for healthy animals. In general, arthritic animals had greater *V*_*VOI*_ at each time point compared to healthy animals. *V*_*VOI*_ of arthritic animals was calculated for various intensity thresholds in the range of 80% to 120% of the threshold calculated by Otsu’s method, which was used to segment the VOI. The variation of the threshold had no impact on the increase of *V*_*VOI*_ of arthritic animals at day 18 after immunization (Supplementary Fig. S9).

### Correlation of quantitative measurements of experimental arthritis severity

**Figure 8:**
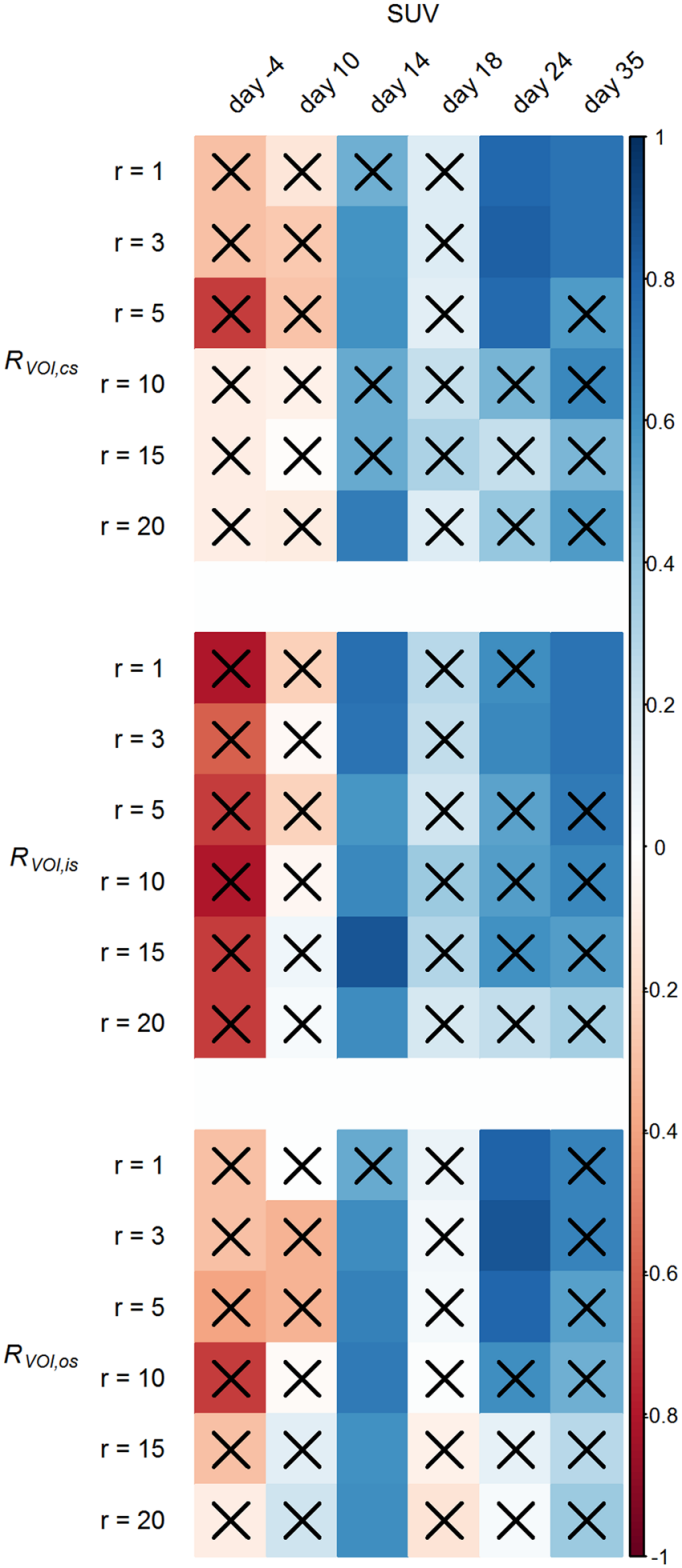
Correlation matrix for PET SUV and VOI roughness for different roughness radii *r* and time points. Statistically insignificant correlations are marked with a black X. The direction of correlation is identified by a color gradient, ranging from red color (negative correlation) to blue color (positive correlation). Statistically significant correlations between SUV and VOI roughness were found at days 14, 24 and 35 after immunization, while at days 24 and 35 only small roughness radii *r* ranging from 1 to 5 yielded significance.

We performed a correlation analysis for the results of clinical scoring, PET measurements and CT roughness values. To test the correlation between clinical score and PET SUV we used the cumulative clinical scores from fore and hind paws of arthritic animals at days 10 to 35 after immunization. Statistically significant correlations were found at days 10 and 14 with *ρ* = 0.87 (*p* < 0.001) and *ρ* = 0.59 (*p* = 0.002), respectively. For correlation between clinical score and VOI roughness we only considered the cumulative clinical scores of the hind paws of arthritic mice at days 10 to 35 after immunization, because roughness analysis was only carried out for the hind paws. We found statistically significant correlations between the clinical score and *R*_*VOI,is*_ for roughness radii *r* = 1 and *r* = 10 (*ρ* = 0.65 with *p* = 0.032 and *ρ* = 0.65 with *p* = 0.032) and *R*_*VOI,os*_ for *r* = 10 (*ρ* = 0.69 with *p* = 0.018) at day 14 after immunization. For the correlation analysis between PET and CT we used the SUVs and VOI roughness values of arthritic and healthy animals from all investigated time points. We found positive correlations between SUV and *R*_*VOI,cs*_, *R*_*VOI,is*_ as well as *R*_*VOI,os*_ for different roughness radii at days 14, 24 and 35 after immunization (Fig. 8).

## Discussion

In this study we examined the feasibility of combined *in vivo* PET/CT imaging to quantify metabolic and anatomic changes in bones caused by experimental arthritis in mice. The immunization of DBA/1 mice with G6PI induced a symmetrical polyarthritis that was marked by swelling and redness of the paws. The onset of the disease could visually be detected at day 9 after immunization and longitudinal imaging was started at day 10 to ensure unimpeded manifestation of arthritic processes.

Already in the early acute stage of the disease arthritic animals showed significantly increased uptake of [^18^F]-fluoride, whereby enhanced tracer uptake could be first observed in the hind paws. After a peak at day 24 after immunization the uptake of [^18^F]-fluoride declined. These results are consistent with earlier findings^10^ and demonstrate that static PET imaging with [^18^F]-fluoride after a short incubation time of 35 min is able to capture pathological bone turnover in experimental arthritis. Furthermore, untreated control animals showed increased uptake of [^18^F]-fluoride in the hind paws towards the end of the experiment. Chronic stressful conditions can cause alterations of bone turnover, associated with bone loss and osteoporosis^30^, leading to increased mineral-binding capacity. Therefore the increased tracer uptake in control animals can, for example, be explained by mental and physical stress due to repeated narcosis and injection procedures. Nevertheless, the tracer uptake was constantly higher for arthritic animals compared to controls.

Bone erosion is a major characteristic of RA in humans as well as in experimental arthritis models and is often used to grade the severity of the disease^31^. Many preclinical studies aimed to quantify the size of such erosions, either manually or in an automated fashion^5, 16, 32^. So far, only few studies focused on the bone surface roughness, which should be a much more sensitive measure as it is a precursor of measurable erosions of the bone^17, 33, 34^.

We quantified the roughness of bony structures of the entire hind paws of mice and called this roughness the VOI roughness, because several factors, such as CT image resolution, bones in close vicinity and the partial volume effect, make it impossible to find the true boundary between bone and surrounding tissue. Our VOIs are a representation of the bony structures which may contain dense material that is in close proximity to the real bones surface.

Our image analysis pipeline of µCT images is completely automatic and comprises three main steps: (i) automated preparation of VOIs, (ii) reconstruction of 3D VOI surface meshes and (iii) the calculation of VOI roughness. The automated fashion of our image analysis pipeline ensures that the quantification of VOI roughness is fast, free of user bias and easily applicable also without expert knowledge in image segmentation or mouse anatomy. Nevertheless, in a few cases the first step produced faulty VOIs, which was mostly caused by untypical projections of the paws or motion artifacts. Therefore, the user has to evaluate the proper VOI representation and to decide if datasets have to be excluded from further analysis, for example, images showing motion artifacts. We made the observation that especially after repeated narcosis within a short period of time it became difficult to maintain a stable depth of narcosis for individual animals. To ensure animal welfare, we preferred less deep narcosis in these cases, accepting an increased number of images containing motion artifacts towards the end of the experiment.

For the first time, we evaluated the progression of bone surface roughness at periosteal and endosteal sites of the cortical bone and resolved the spatial scale on which this roughness occurs. Therefore we calculated the VOI roughness for three different regions (complete cortical bone surface, outer cortical bone surface and inner cortical bone surface) and different roughness radii *r* ranging from *r* = 1 to *r* = 20. The VOI roughness of arthritic animals was significantly increased for all regions already at day 10 after immunization, but periosteal and endosteal sites of the bones showed differences with respect to *r*. While endosteal roughness of arthritic animals is increased for nearly all *r*, the periosteal roughness could only be detected between *r* = 1 and *r* = 5 in the earlier phase of the disease and only at a scale of *r* = 5 to *r* = 20 in the late remitting phase. In general, the roughness occurred on a larger spatial scale on the endosteal surface and is also more pronounced there compared to the periosteal surface. In both cases the roughness reaches its maximum at day 18 and then declines at later time points. These results suggest that bone erosion is not uniformly distributed over the bones surfaces and that the roughness measurements proposed here are able to capture different aspects of the disease, i.e., bone erosion and bone formation. Several studies could show that bone marrow infiltrates lead to increased bone formation at endosteal surfaces close to erosions^26, 27^. In addition, the osteoclastic activity seems to be increased at periosteal surfaces compared to endosteal surfaces^28^ and in adjuvant-induced arthritis new bone formation was observed at the periosteal bone surface^29^. Taken together, it is reasonable to assume that bone erosion can be measured as roughness on a smaller spatial scale, while bone formation is marked by increased roughness on a larger spatial scale and that these two processes may be evaluated simultaneously by *in vivo* µCT imaging. This would be of great advantage in drug evaluation studies that are focused on reduction of erosive processes and induction of bone repair as the quantitative methodology proposed here is non-invasive and can be applied longitudinally without the need to sacrifice test animals at each measurement time point. However, to prove this hypothesis more investigations are required in the future and the correlation between bone surface roughness, the scales on which it occurs and the processes of bone erosion and formation have to be evaluated first. This includes histological analysis and should be part of future studies on experimental arthritis models.

In an earlier study the quantitative assessment of PET tracer uptake was clearly superior of µCT-based measurements with regard to detecting early alterations of the bones in experimental arthritis^10^. PET imaging would therefore be the means of choice when sensitivity is indispensable, even though it does cause additional costs, a logistic overhead and additional radiation dose compared to CT imaging alone. In contrast, our results demonstrate that VOI roughness is much more sensitive to detect early bone erosion than bone volume or surface area measurements, which have been performed previously^10^ and which have frequently been used to quantify arthritic processes in animal models^13, 14, 32, 35, 36^. In fact, we found no delay between the onset of increased PET tracer uptake and increased bone surface roughness after arthritis induction, emphasizing the capability of bone surface roughness analysis to quantify pathological alterations of the bones immediately after arthritis onset. However, our correlation analysis showed that PET SUV and VOI roughness are not necessarily correlated throughout the course of experimental arthritis (see Fig. 8). Statistically significant correlations were only found at days 14, 24 and 35 after immunization. At the onset of arthritis manifestation at day 10 as well as at the peak of bone surface roughness at day 18 the two measurements did not correlate with each other. At days 24 and 35, only statistically significant correlations were found for small roughness radii *r* in the range of 1 to 5. This observation is further supporting our hypothesis that bone erosion can be measured as roughness on a small spatial scale because the erosion of the bone causes an increasing surface area and is therefore directly connected to the mineral-binding capacity of the surface and also to the uptake of [^18^F]-fluoride. While PET SUV and VOI roughness were mainly correlated at the later stages of the disease, statistically significant correlations between PET SUV and clinical score or VOI roughness and clinical score could only be found in the early acute stage at days 10 and 14. This result indicates that macroscopical signs of experimental arthritis are closely connected to the onset of arthritic processes but do not represent the severity of bone surface roughness and pathological bone turnover.

An important therapeutic aim in the treatment of arthritis patients is to stop bone erosion or overshooting. To assess the efficacy of new drugs, sensitive diagnostic methods are needed and combined PET/CT imaging as shown here would assist this process by providing a non-invasive tool that allows the quantification of bone metabolism, bone erosion and formation. While µCT-based assessment of bone surface roughness turned out to be very sensitive towards early alterations of the bones, our correlation analysis indicates that PET and CT imaging capture different aspects of the disease and may augment the knowledge gained in experimental studies when used in combination.

## Electronic supplementary material

Supplement
