# Supplementary material for: Automated Quantification of Early Bone Alterations and Pathological Bone Turnover in Experimental Arthritis by in vivo PET/CT Imaging
Source: Sci Rep. 2017 May 22;7:2217. doi: 10.1038/s41598-017-02389-6 (PMC5440413; doi:10.1038/s41598-017-02389-6)
Supplement: Supplementary file 1 — Supplement [file 41598_2017_2389_MOESM1_ESM.pdf]

Supplementary Material

Automated Quantification of Early Bone Alterations and Pathological Bone Turnover in Experimental Arthritis by *in vivo* PET/CT Imaging

Bianca Hoffmann[a,b,1], Carl-Magnus Svensson[c,1], Maria Straßburger[d], Björn Gebser[a], Ingo M. Irmler[e], Thomas Kamradt[e], Hans Peter Saluz[a,b,*], Marc Thilo Figge[b,c,*]

Topic	Page
• Macroscopical scoring of arthritis severity (Figure S1)	2
• Placement of PET VOIs around fore and hind paws (Figure S2)	3
• Endpoint determination for automated preparation of VOIs based on µCT images (Figure S3)	4
• Impact of roughness radius *r* (Figure S4)	5
• Estimation of goodness of fit for gamma probability density function and lognormal probability density function (Figures S5, S6 and S7)	6
• Overview on study design and image analysis pipeline (Figure S8)	9
• Robustness of µCT-based assessment of VOI volume (Figure S9)	10
• Number of data points used for statistical analysis (Table S1)	11
• References	11

Macroscopical scoring of arthritis severity

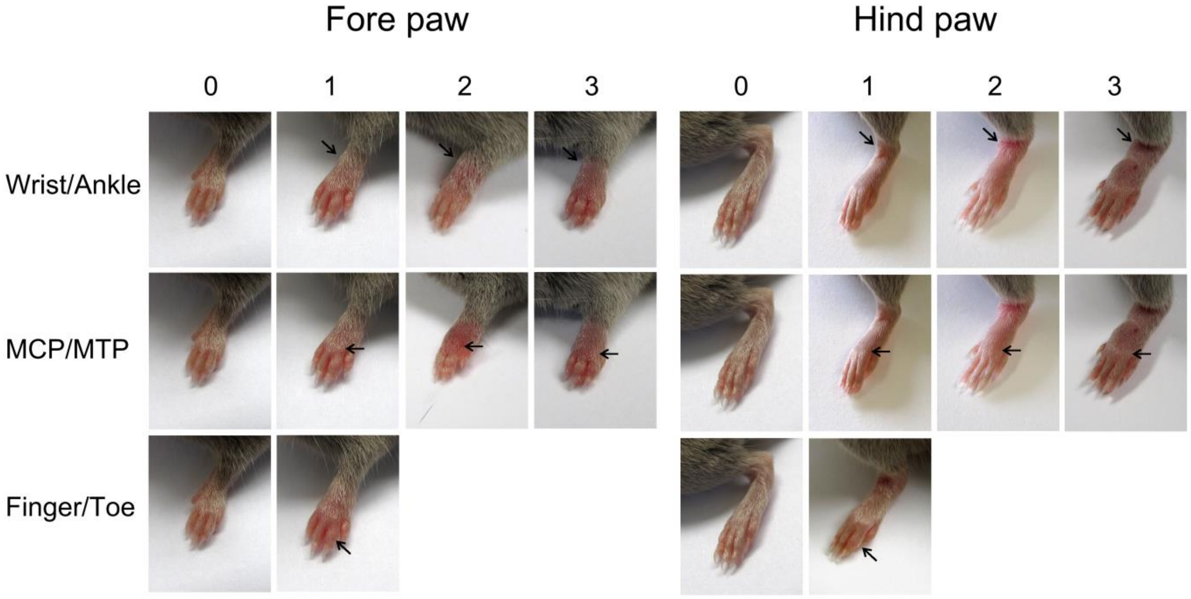

Figure S1: Macroscopical scoring of arthritis severity. According to the degree of swelling and redness scores between 0 and 3 are assigned to the wrist/ankle region, the metacarpophalangeal (MCP)/metatarsophalangeal (MTP) region and the fingers/toes of each paw. The score for fingers/toes is half of the number of swollen fingers or toes of one paw.

Placement of PET VOIs around fore and hind paws

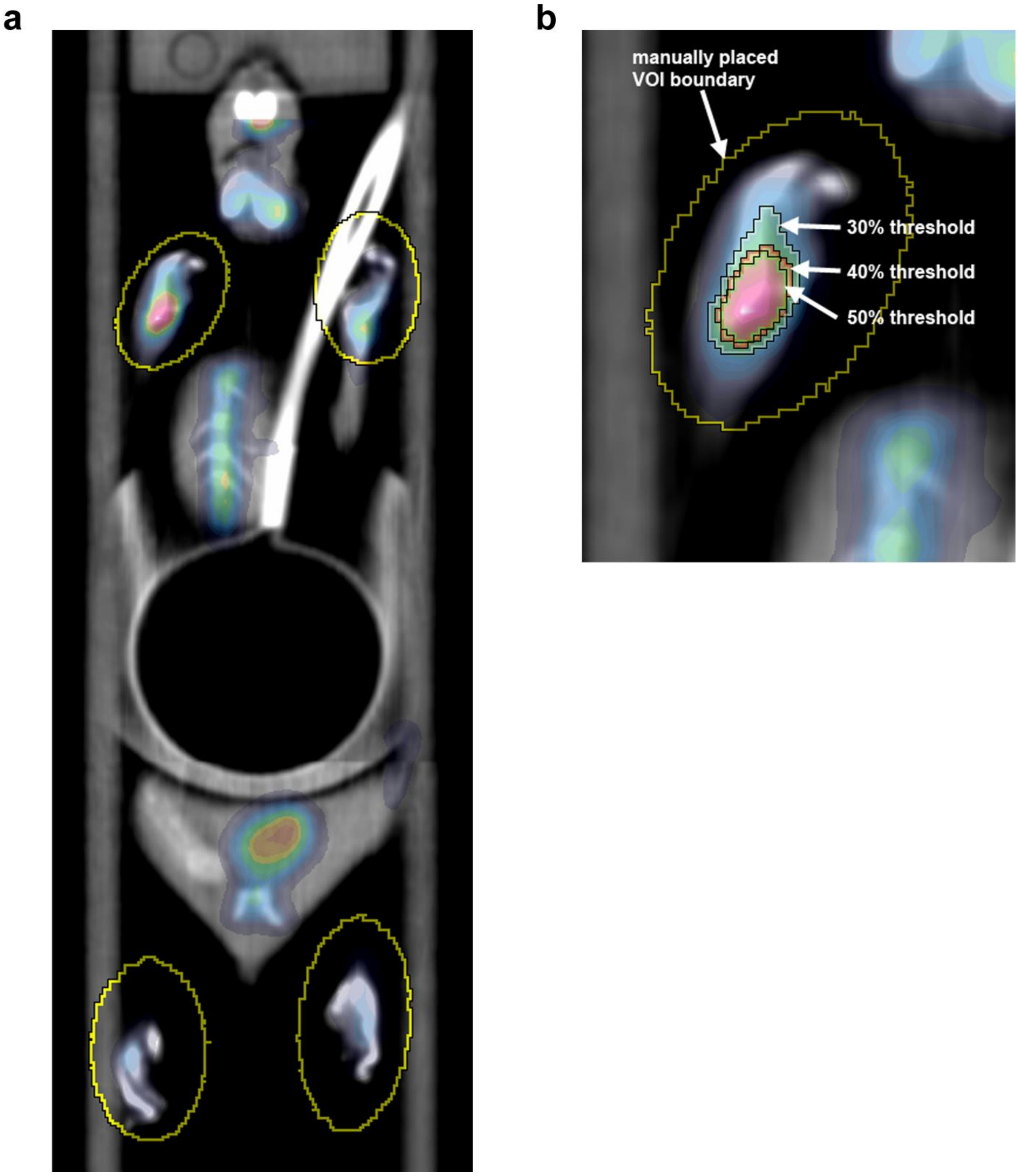

Figure S2: Placement of PET VOIs around fore and hind paws and SUV VOI thresholding. **(a)** Guided by the CT images 3d ellipsoid VOIs were placed manually around fore and hind paws in a way that all bone and joint structures are captured. The VOIs are shown here as yellow color in a cross-sectional coronal slice of fused PET (color) and CT (grey) images. **(b)** Reasonable SUV threshold values are ranging from approximately 30% to 50%, while this is not a very sensitive parameter as can be seen by the close outlines of the different thresholds in the figure.

Endpoint determination for automated preparation of VOIs based on µCT images

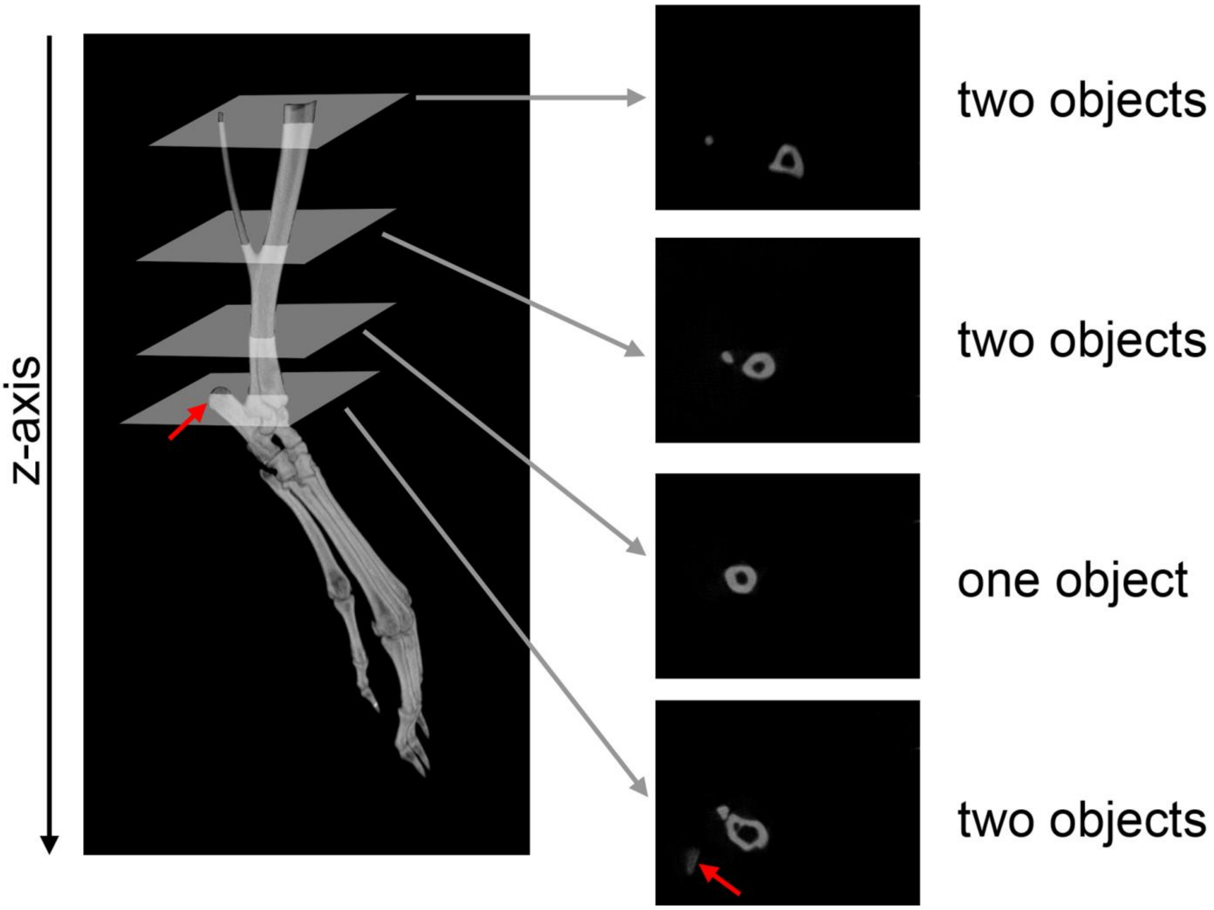

Figure S3: Endpoint determination for automated preparation of VOIs based on µCT images. The endpoint of the hind paws is defined as the point where the tibia enters the tarsocrural joint and the calcaneus (red arrows) starts to appear in the image stack. This position is found by the typical pattern of the number of objects that are visible in the cross-sectional slices in z-direction. First, tibia and fibula are visible (two objects) which are then fusing to one visible object. As soon as the calcaneus starts to appear in the image stack, again two objects can be detected and the endpoint of the hind paw is found.

Impact of roughness radius *r*

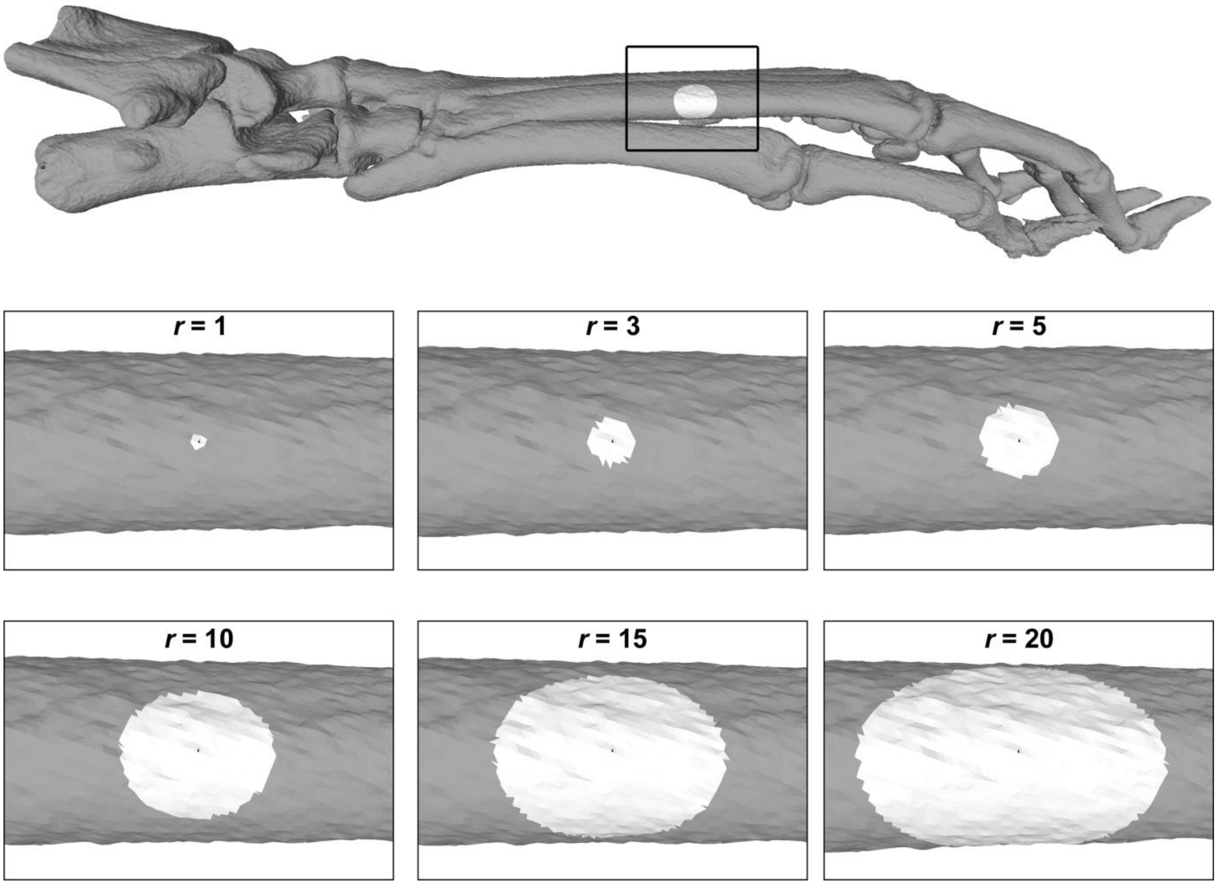

Figure S4: Visualization of the roughness radius *r*. It defines the size of the circular vicinity around one triangular facet of the mesh in which the local roughness for this facet is calculated. The example above shows this vicinity (white color) for one facet (black color) and different roughness radii *r* between 1 and 20.

Estimation of goodness of fit for gamma probability density function and lognormal probability density function

The shape of the composite histogram of all observed angles in the control data (before immunization) very much depends on the VOI and the roughness radius *r*. Silva *et al.* proposed to use a gamma probability density function (PDE) as an approximation of the angle distribution [1]. In our data analysis we found that often the lognormal PDE yields a better representation of the shape of the composite histogram than the gamma PDE (Figure S4). The goodness of fit of both PDEs was measured quantitatively by the Kullback-Leibler (KL)-divergence which is defined as

$$D_{KL}(p \| q) = \sum_{x} p(x) \log \frac{p(x)}{q(x)} \tag{1}$$

where *p(x)* and *q(x)* are two probability distributions over the discrete random variable *X*. The KL-divergence was significantly lower for the lognormal PDE than for the gamma PDE (Figure S5), therefore we decided to approximate the angle distribution by the lognormal PDE. Significance was tested by Wilcoxon rank sum test. The effect on the calculated threshold is normally small in terms of the cutoff angle, as can be seen in the examples in Fig. S4. The effect on the calculated threshold is normally small in terms of cutoff angle, see examples in Figure S5. The threshold determined by using the gamma fit is denoted T_{gamma}, and consequently the threshold determined by using the lognormal fit is denoted $T_{lognorm}$. We denote the percentages of angles that are larger than a threshold, T, as $P(T)$ and compare the differences between the percentages by taking $P(T_{lognorm}) - P(T_{gamma})$. In Fig. S6, we show a histogram describing the percentage point (p.p.) difference for fits to 324 angle distributions. For the majority of fits a change of distribution leads to a change of less than 1% of the number of angles used for the roughness calculation. The percentage of angles considered for roughness analysis is normally around $P(T) \approx 5\%$ for both fits.

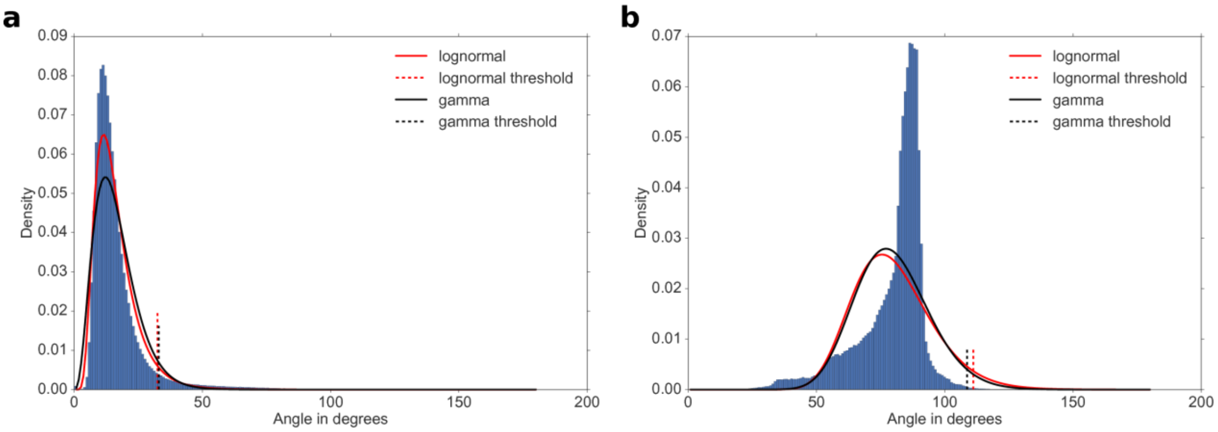

Figure S5: Lognormal and gamma distributions fitted to two representative angle histograms. The distribution of angles in one paw depends on the VOI as well as the roughness radius *r*. While usually the lognormal distribution function fits better to the density histogram than the gamma distribution function **(a)** there are also cases where the latter fits better **(b)**.

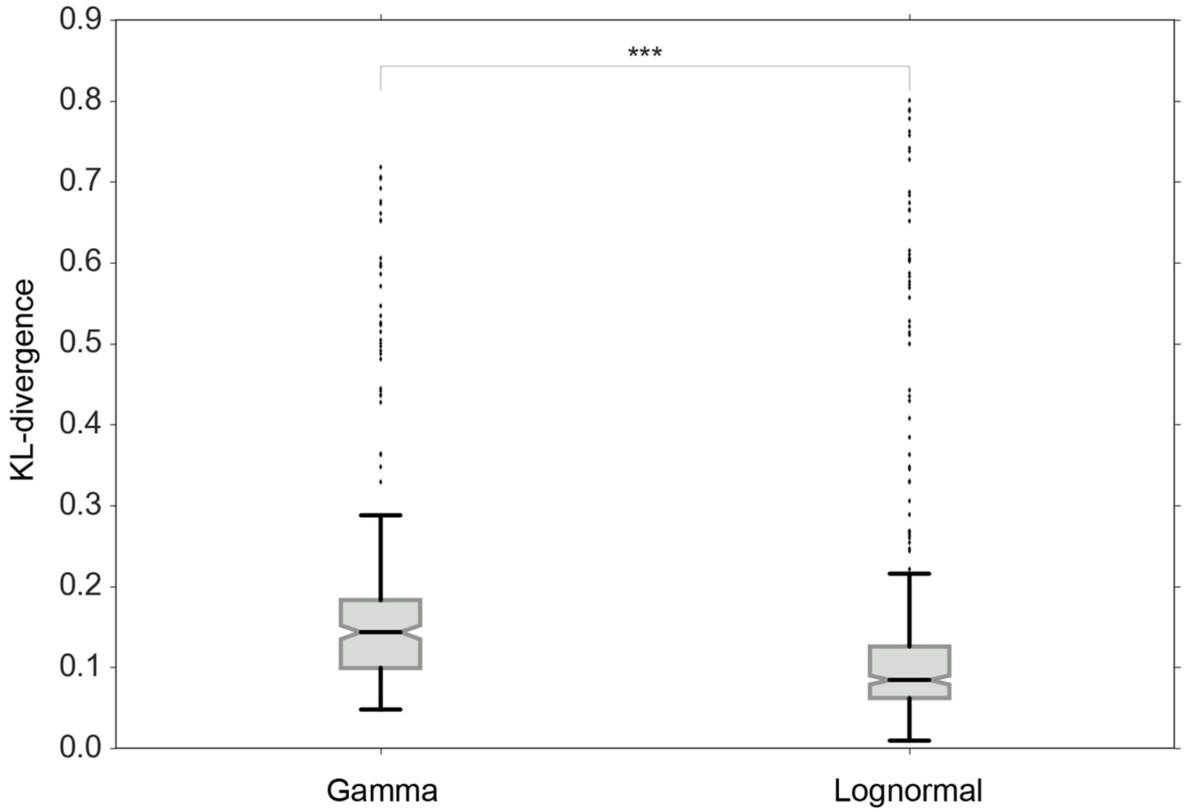

Figure S6: Estimation of goodness of fit for gamma and lognormal distribution functions. The Kullback-Leibler divergence is significantly smaller for the lognormal distribution function fitted to histograms of angles compared to the gamma distribution function ($p < 10^{-13}$, N = 324).

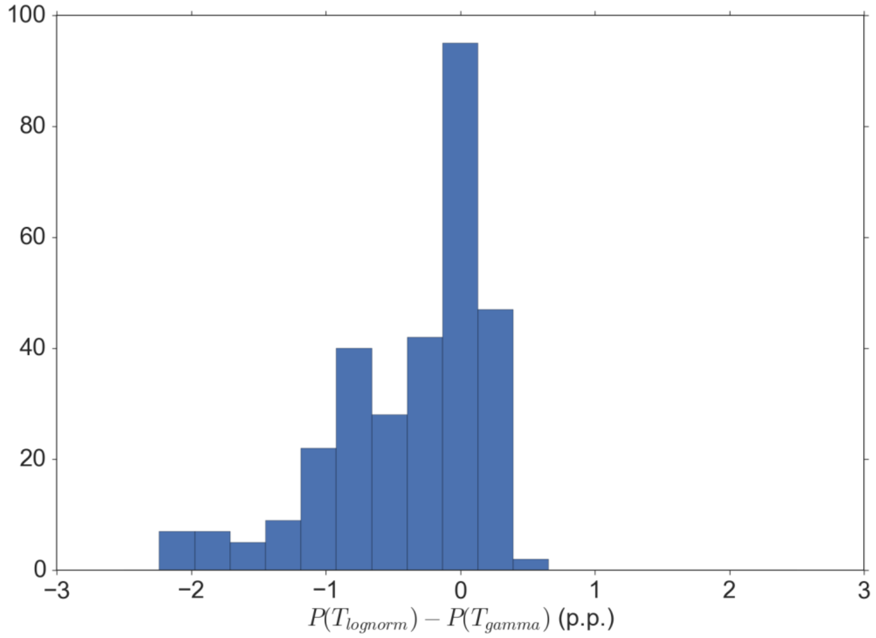

Figure S7: The percentage point (p.p.) difference of angles used for roughness calculation between thresholds based on lognorm fitting and gamma fitting. The histogram is based on fits to 324 angle distributions.

Overview on study design and image analysis pipeline

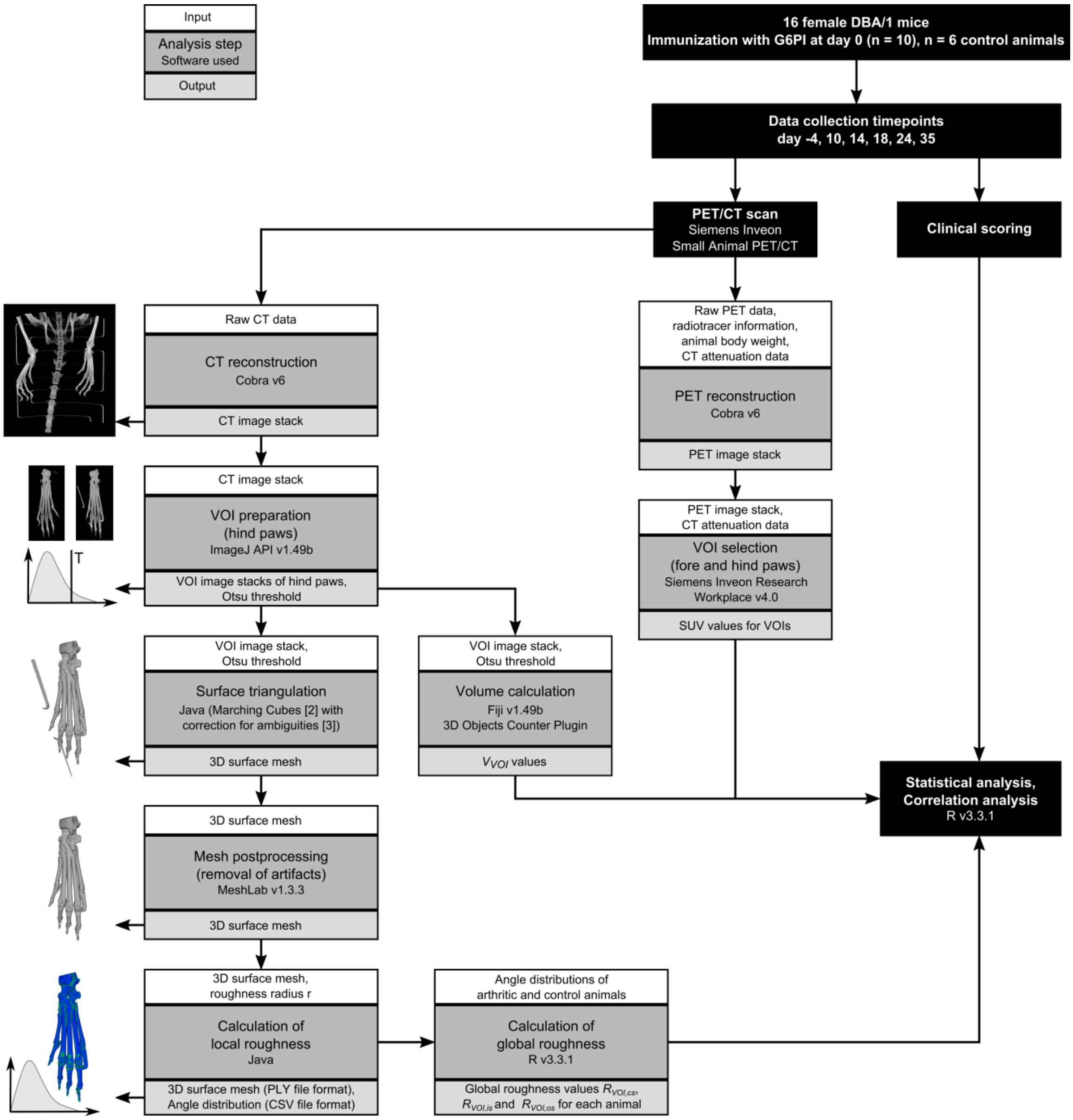

Figure S8: Overview on study design and image analysis pipeline. 10 female DBA/1 mice were immunized with G6PI at day 0 and 6 mice were used as non-immunized controls. Imaging and clinical scoring were performed longitudinally before immunization and at different time points of acute and chronic arthritis. The acquired PET data was used to calculate the [^{18}F]-fluoride SUV of fore and hind paws as a measure of bone metabolism. Based on the µCT data VOIs were prepared which included all bone and joint structures of the animals hind paws. For later comparison we prepared VOIs for the complete bone surface, the inner bone surface and the outer bone surface. 3d surface representations of these VOIs were then constructed by surface triangulation and artifacts were removed in a mesh postprocessing step. The local roughness for each facet of a mesh was calculated for different roughness radii *r* and saved as angle distribution for each paw. In a last step the global roughness for complete ($R_{VOI,cs}$), inner ($R_{VOI,is}$) and outer ($R_{VOI,os}$) surface was calculated for each paw.

Robustness of µCT-based assessment of VOI volume

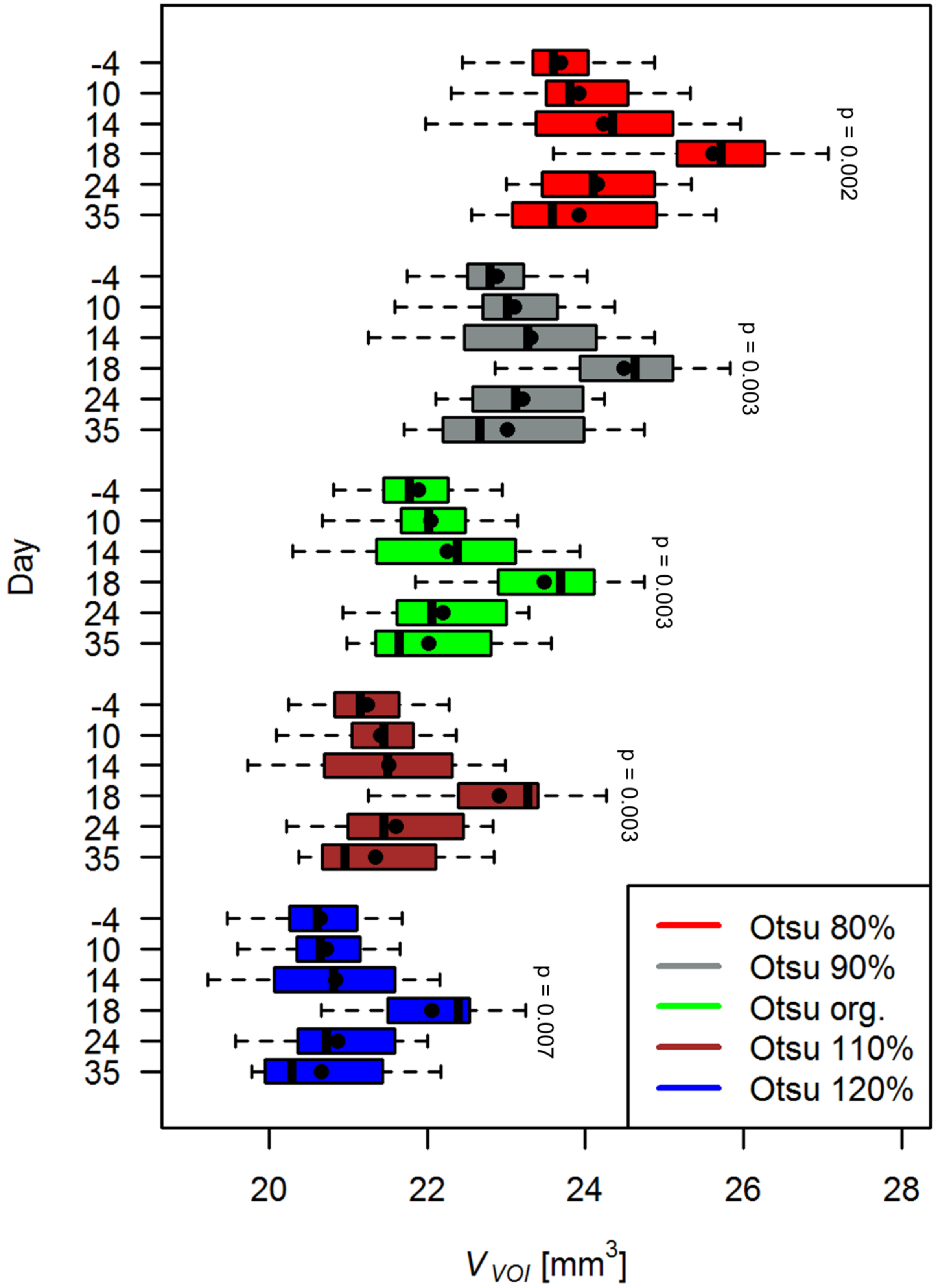

Figure S9: VOI volume for arthritic animals and different intensity thresholds. The increased VOI volume for arthritic animals at day 18 after immunization is not influenced by variation of the intensity threshold between 80% and 120% of the threshold calculated by Otsu's method.

Number of data points used for statistical analysis

Table S1: Number of data points used for statistical analysis (AR – arthritic; CO – control).

	Day					
**Experiment**	**-4**	**10**	**14**	**18**	**24**	**35**
PET<br># fore paws (AR/CO)<br># hind paws (AR/CO)	 10<br>10	 6/12<br>6/12	 12/8<br>12/8	 14/10<br>14/10	 14/10<br>14/10	 10/10<br>10/10
CT<br># hind paws (AR/CO)	10/9	15/12	12/10	8/9	9/8	8/5
Clinical Score<br># animals	-	10	10	9	7	5

References

[1] Silva, M. D. *et al.* Application of surface roughness analysis on micro-computed tomographic images of bone erosion: examples using a rodent model of rheumatoid arthritis. *Mol. Imaging* **5,** 475–484 (2006).

[2] Lorensen, W. E. & Cline, H. E. Marching cubes: A high resolution 3D surface construction algorithm. *ACM SIGGRAPH Comput. Graph.* **21,** 163–169 (1987).

[3] Cignoni, P., Ganovelli, F., Montani, C. & Scopigno, R. Reconstruction of topologically correct and adaptive trilinear isosurfaces. *Comput. Graph.* **24,** 399–418 (2000).
